# Heredity as a factor in the formation of recurrent depression

**DOI:** 10.1192/j.eurpsy.2022.390

**Published:** 2022-09-01

**Authors:** N. Maruta, S. Kolyadko, T. Panko, O. Semikina

**Affiliations:** “Institute of Neurology, Psychiatry and Narcology of NAMS of Ukraine” SI, Borderline Pathology, Kharkiv, Ukraine

## Abstract

**Introduction:**

At the current stage of psychiatry development, special attention is paid to studying the influence of hereditary factors on the occurrence of recurrent depression (RD). The study can be informative in predicting the risk of the RD occurrence RD. Therefore, studies related to this problem are designed to identify the specificity “familial” forms of RD.

**Objectives:**

To study the influence of hereditary factors on the RD formation.

**Methods:**

Clinical-psychopathological, clinical-genealogical, statistical.

**Results:**

Based on the clinical and genealogical data study, a statistically significant excess of the individuals with psychiatric disorders proportion in the main group (108 patients with RDD whose family history included relatives with depression, main group) was found: The percentage of individuals on psychiatric registry (18%, CI: 14.5-22.1) was 15 times higher than the control group (46 individuals without RDR in the pedigree) (p<0.05), individuals with depression (33%, CI: 28.5-37.8) were 7.3 times higher (p < 0.05), suicides (7.9%, CI: 5.6-11.0) were 4.2 times higher (p < 0.05), cases of alcohol dependence (25.6%, CI: 21.6-30.2) were 1.8 times higher (p < 0.05). In the main group family tree examinees, this pathology occurred most frequently in I and II degree of kinship relative. When comparing heredity factors with peculiarities of the RD course, we found a specific weight in correlations of such factors as: depressive disorders predominantly in first-degree relatives (p ≤ 0.005), suicidal behavior in first- and second-degree relatives (p ≤ 0.005).

**Conclusions:**

The findings should be taken into account in diagnostic and preventive measures.

**Disclosure:**

No significant relationships.

